# Macronutrients in Parenteral Nutrition: Amino Acids

**DOI:** 10.3390/nu12030772

**Published:** 2020-03-14

**Authors:** Roberto Iacone, Clelia Scanzano, Lidia Santarpia, Iolanda Cioffi, Franco Contaldo, Fabrizio Pasanisi

**Affiliations:** Clinical Nutrition Unit, Department of Clinical Medicine and Surgery Federico II University Hospital, 80131 Naples, Italy; clelia.scanzano@unina.it (C.S.); lidia.santarpia@unina.it (L.S.); iolanda.cioffi@unina.it (I.C.); contaldo@ds.unina.it (F.C.); pasanisi@unina.it (F.P.)

**Keywords:** amino acid, parenteral nutrition, essential amino acid, amino acid requirement, nitrogen balance, energy requirement

## Abstract

The right amount and quality of amino acids (AAs) supplied to patients on parenteral nutrition (PN) reduces muscle mass loss, may preserve or even increase it, with significant clinical benefits. Several industrial PN mixtures are available so that nutrition specialists can choose the product closest to the patient’s needs. In selected cases, there is the possibility of personalizing compounded mixtures in a hospital pharmacy that completely meets the individual nutritional needs of PN patients. This narrative review deals with the AA solutions used in PN mixtures. The physiology, the methods to calculate the AA needs, and the AA and energy requirements suggested by scientific guidelines for each patient type are also reported.

## 1. Introduction

Parenteral nutrition (PN) is a complex medical therapy used when the gastrointestinal (GI) system is not working or unavailable for use [1]. The main PN indication is for patients with intestinal failure (short bowel syndrome, inflammatory bowel diseases, intestinal pseudo-obstruction, radiation enteritis), high-output intestinal fistulas, severe intestinal obstruction, and in preterm infants with a not-yet-fully developed GI apparatus [2]. PN also has high relevance in treating malnutrition and prevents complications during the perioperative period [3,4]. Indeed, pre-surgery and post-surgery PN (7–10 days before and/or after surgery) improves the postoperative outcome in patients with severe malnutrition, who are unable to be fed orally/enterally [5,6,7,8]. PN is also used in cancer patients with side effects (mucositis, stomatitis, esophagitis) related to chemo- or radiotherapy toxicity significantly influencing oral/enteral food intake for more than seven days [9,10,11], and in general to prevent and treat malnutrition that would lead to treatment interruption/delay with a negative influence on individual prognosis [12]. Finally, PN is lifesaving in critically ill patients with mesenteric ischemia, intestinal hemorrhage, intractable diarrhea, uncontrollable vomiting, paralytic ileus, peritonitis, and severe pancreatitis. As to PN duration, it can be lifelong (and lifesaving) in patients with chronic intestinal insufficiency, as well as temporary or palliative in other clinical conditions.

In PN, nutrients are administered intravenously; in a more foundational building-block state, through a peripheral or central vein. The preparation of the infusion mixture utilizes sterile solutions of amino acids (AAs), glucose, lipids, water, electrolytes, trace elements, and vitamins. PN mixtures are defined as "standard" if industrially prepared with a fixed composition and pre-packaged (or otherwise known as “ready-to-use”) or "personalized" if compounded in a hospital pharmacy for the specific nutritional needs of the single patient [13] and patients with particular organ failure (i.e., volume restriction in heart failure, electrolyte balance in chronic renal failure); furthermore, personalized PN mixtures are often required for critically ill and/or severely catabolic patients, as for patients with benign chronic intestinal insufficiency and preterm born infants [13]. This narrative review deals with the AA solutions used in PN mixtures. The physiology, the methods to calculate the AA needs, and the AA and energy requirements suggested by scientific guidelines for patients’ type are also reported.

## 2. Amino Acids: Physiology

AAs are the structural units of proteins, key elements for the life-sustaining chemical processes. Proteins can have a structural role (constituting muscles, organs, glands, ligaments, tendons, nails, hair, bones) or can be hormones and enzymes catalyzing and regulating most of the vital processes in the body. Some AAs (named functional AAs: arginine, cysteine, glutamine, leucine, proline, and tryptophan) regulate key metabolic pathways, fundamental for immunity, reproduction, maintenance, and growth [14]. Proteins and AAs also represent the only source of nitrogen for humans. Since the body has no protein storage, the body’s growth, cellular structure preservation, wound healing, enzymatic, and hormonal heritage depend on the constant quantity and quality of external proteins (or AAs) to compensate daily losses. Twenty AAs differently combined, form proteins; nine AAs (leucine, isoleucine, valine, lysine, phenylalanine, histidine, methionine, threonine, and tryptophan) are essential AAs (EAAs) and are indispensable since they cannot be synthesized de novo in physiologically significant quantities; therefore, they must be provided in adequate amounts from the outside. Out of the other eleven AAs, some are considered conditionally essential AAs (CEAAs, e.g., cysteine, tyrosine, arginine, proline, glycine, serine, and glutamine), as they may become essential under certain circumstances. Furthermore, taurine should also be added. From the chemical point of view, taurine is not an amino acid but rather an amino-sulphonic acid; it is deficient in premature infants lacking the enzyme converting cystathionine into cysteine [15]. The body can produce some AAs (alanine, asparagine, aspartic acid, glutamic acid), which are therefore considered non-essential AAs (NEAAs). However, NEAAs must be in adequate quantities together with EAAs and CEAAs to achieve protein synthesis.

**Figure 1:**
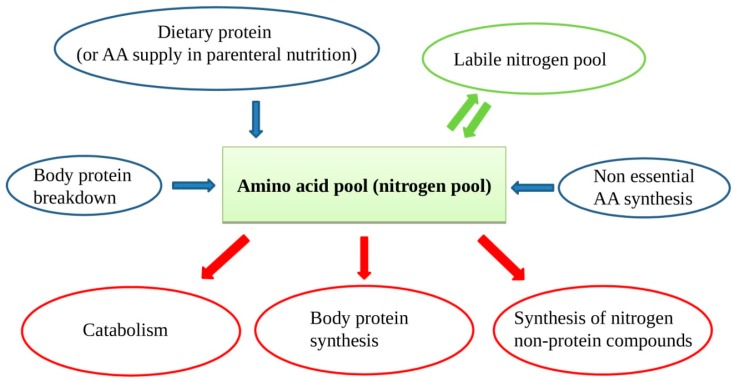
The amino acid (AA) pool contains AAs arising from dietary source, protein breakdown, non-essential AAs (NEAAs) from liver synthesis, and labile nitrogen pool. As the body cannot store proteins, there is a continuous AA turnover with a constant AA input balancing the AA output.

In patients on PN, protein intake is met by supplying intravenous sterile free AA solutions. In case of chronic lack of one or more AAs, some protein synthesis, and consequently vital functions, could be compromised. In the case of inadequate supply, muscle AAs will be used, with consequent muscle mass reduction and atrophy. Muscle proteins are in constant turnover with continuous new protein synthesis and breakdown. The AAs arising from protein breakdown and those supplied from outside form the so-called "nitrogen or amino acid pool", a large mixture of all the available AAs for new protein synthesis, nitrogen non-protein molecules, and energy production (Figure 1). Besides, when the external provision of AA is in lack for some time, a portion of AA can be supplied by the so-called labile nitrogen pool. The labile nitrogen pool or labile protein represents a phenomenon of rapid gain or loss of body proteins in response to changes in protein intake [16]. If a healthy adult switches from a protein-rich diet to a protein-free diet, urinary nitrogen excretion remains considerable for a few days, before decreasing to the steady-state of obligatory nitrogen loss. This represents a loss from the body of approximately 40 g of nitrogen or 250 g of rapidly mobilizable protein. The labile protein pool mainly resides in splanchnic tissues.

**Figure 2:**
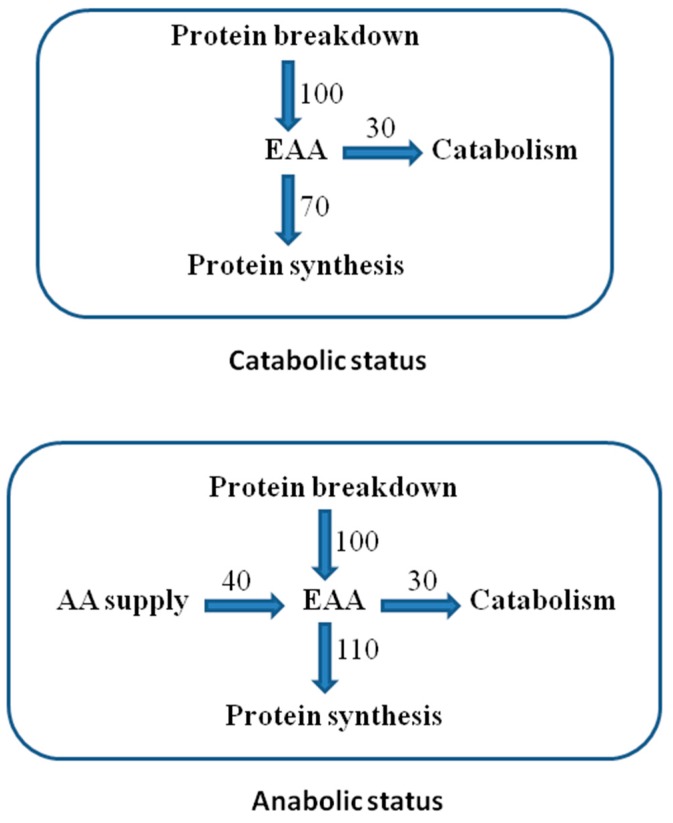
Essential AA (EAA) recycling in protein turnover. Reported units are arbitrary to explain the differences between the anabolic and catabolic states in the presence or absence of external AA supply.

Adequate availability of all EAAs (supplied and from protein breakdown) enhances protein synthesis, determining an anabolic state [17] (Figure 2).

It is essential to consider that, although necessary for protein synthesis, NEAAs cannot act as a trigger, differently from EAAs. For this reason, the effectiveness of a given nitrogen intake is linked to the EAA, rather than to the total AA content [18]. Moreover, an excessive NEAA supply (without an adequate EAA quote) would exclusively increase catabolism.

## 3. Amino Acid Solutions for Parenteral Nutrition

Muscle atrophy (rather difficult to reverse) consisting of striated-fiber muscle depletion causes weakness, loss of motor function up to sarcopenia, and cachexia [19]. Muscle mass preservation is one of the main PN goals; the intravenous infusion of AA solutions preserves a positive nitrogen balance and promote muscle protein synthesis. Standard AA solutions for PN contain EAAs, some NEAAs (to reach the required quantity of nitrogen), and occasionally some CEAAs. In specific clinical conditions, such as AA disorders (e.g., inborn errors in the metabolism of some AAs), preterm born, and critically ill patients, specialized AA solutions may be required [20]. In recent work, we showed that commercially available standard PN mixtures, even with the same total AA content, have very different EAA/AA ratios, and in particular, an unlikely EAA profile (Table 1 and Table 2, and [21]).

Although all evaluated mixtures at the usual dose of 1 g/kg of body weight per day meet the EAA doses for unstressed healthy subjects suggested by the World Health Organization (WHO), the Food and Agriculture Organization of the United Nations (FAO), and the United Nations University (UNU), in most cases their EAA content was insufficient to guarantee a significant muscle mass increase in patients on PN; thus, to obtain an effective dose of EAA, it would be necessary to increase the daily dose of total AAs considerably. In the AA mixtures for PN, the EAAs should be at least 50% of the total AA support and, in particular, leucine, isoleucine, and methionine content should exceed a fixed threshold to obtain a gain of muscle mass in PN patients [21]. 

The ideal AA solution able to satisfy the nutritional needs of all patients requiring PN does not exist; indeed, very often, it must be personalized according to the single needs. Recently, the presence of two NEAAs (tyrosine and cysteine) in the EAA mixtures seem to be crucial [22]. Tyrosine is an NEAA only for the liver (and partially for the kidney), while it is an EAA for all other organs/tissues lacking phenylalanine hydroxylase, the enzyme converting phenylalanine into tyrosine. For this reason, the addition of tyrosine to other EAAs would improve the effectiveness of tyrosine synthesis.

Several dietary AAs are metabolized or converted into other ones within the intestine or liver on the first pass. Animal studies suggest that the intestine primarily uses food-derived AAs for specific protein synthesis, instead of those provided by systemic circulation [23]. By PN, the intestine is bypassed with the consequent reduced systemic availability of some AAs, finally resulting in their increased parenteral requirement. Thus, it appears that parenterally administered phenylalanine and methionine are converted to tyrosine and cysteine, respectively, to a lesser extent than those ingested. In particular, patients with renal insufficiency and prematurely born infants would seem to need tyrosine-enriched mixtures. Chronic renal failure is associated with the loss of the renal activity of phenylalanine hydroxylase, therefore explaining the reduced conversion of phenylalanine into tyrosine [24]. For prematurely born infants, the immaturity of the enzyme system makes it necessary to add tyrosine to the mixtures for PN [25]. Unfortunately, tyrosine has poor water solubility; consequently, the amount added in the PN mixtures is limited. 

Cysteine added to EAA mixtures significantly reduces the toxicity of methionine. Cysteine is synthesized from methionine with the production of homocysteine as a metabolically toxic obliged intermediate. Di Buono et al. [26] suggested that dietary cysteine can reduce the exogenous requirement for methionine in men. These results support the existence of a cysteine sparing effect in humans. Sulfur-containing amino acids are safer when supplied with balanced stoichiometric ratios of methionine and cysteine. In this way, they spere the folate demand and minimize the homocysteine production derived by methionine metabolism to meet cysteine requirements. Cysteine supplementation (in a double dose compared to methionine) into EAA mixtures avoids homocysteine overload.

## 4. Disease-Specific AA Mixture

Concerning the specialized AA mixtures, the study by Plauth et al. [27] suggested the use of standard AA solutions for grade I and II hepatic encephalopathies, and liver-adapted ones from grade III to IV. These latter specialized AA mixtures contain large amounts of branched-chain AAs (BCAAs) and lower doses of aromatic AAs (phenylalanine and tyrosine), methionine, and tryptophan. However, studies on the effectiveness of BCAAs in the treatment of hepatic encephalopathy provided controversial results. Accordingly, the guidelines proposed by the Society of Critical Care Medicine (SCCM) and the American Society for Parenteral and Enteral Nutrition (ASPEN) [28] reported that there is no evidence to suggest that BCAA-enriched formulations improve patient outcomes compared with standard whole-protein ones in critically ill patients with liver disease.

Similarly, Cano et al. in the European Society for Clinical Nutrition and Metabolism ESPEN Guidelines on adult renal failure [29] suggest the use of BCAAs in patients with non-dialyzed renal failure, differently from the SCCM and the ASPEN guidelines. In patients with severe disorders of AA utilization (for example, inborn errors of AA metabolism), special mixtures must be used. For instance, in phenylketonuria (a disease deriving from the lack of phenylalanine hydroxylase, with the inability to transform phenylalanine in tyrosine), AA mixtures without phenylalanine are necessary to avoid phenylalanine overload and brain toxicity [30]. Prematurely born infants need PN mixtures enriched in cysteine, tyrosine, taurine, glutamine, and arginine to overcome the metabolic immaturity and allow a healthy growth [31]. Moreover, in prematurely born infants, the plasma levels of the single AAs need to be strictly monitored and corrected due to the immaturity of protein breakdown enzymes. For adult patients in intensive care units (ICU), complete PN mixtures with a high protein/energy ratio would be recommended [32,33]. Unfortunately, most all-in-one industrialized mixtures generally do not guarantee sufficient AA supply to restore nitrogen losses [22,34,35,36,37]. In these selected cases, personalized all-in-one PN mixtures represent the best solution to satisfy patient needs and prevent possible complications (i.e., overfeeding or refeeding in case of over-calorie supply). 

Intrinsic acidity of AA mixtures is often neglected [38]: metabolic acidosis (in the short term) [39,40] and metabolic bone disease (long term) [41,42] could derive from the excessive exogenous administration and/or massive endogenous production of non-volatile acids. The exogenous non-volatile acids originate from inorganic acids added to the PN mixtures to support their physical–chemical stability. The production of endogenous non-volatile acids mainly derives from the metabolism of some AA (cysteine, methionine, lysine, histidine, arginine). The acid load following PN mixture administration can be corrected, adding the right doses of organic acids or their salts, such as acetate, gluconate, aspartate, or citrate [38].

## 5. Protein Requirement

The requirement for an essential nutrient is the amount of nutrient that satisfies the body’s needs; it corresponds to the metabolic demand obtained by the sum of all metabolic nutrient-consuming pathways. The AA metabolic demand is the AA flow through specific pathways aiming to maintain body structure and function and achieve important metabolites [43]. AA metabolites are then transformed into final nitrogen products such as urea, ammonia, uric acid, creatinine (excreted with urine), and other nitrogen compounds eliminated with feces, sweat, and other excretory tracts, or seized and lost with nails, hair, and skin peeling [44]. The estimated daily of macro and micronutrient requirements rely on epidemiological studies carried out on healthy subjects to prevent deficiency syndromes. Consequently, the minimum recommended nutrient intakes (dietary reference intakes) suggested by WHO, FAO, and UNU do not refer to people with different clinical conditions and therefore represent only a starting point for the calculation of the nutritional requirements for these patients [43]. The most widely used method to establish the daily protein requirement is the nitrogen balance [45]. Briefly, the minimum daily protein (or AA) intake corresponds to the minimum protein requirement to keep a nitrogen equilibrium [46,47], in other words, to balance nitrogen losses in the body by protein intake. This results from two general characteristics that the body has concerning AA turnover: (1) the adaptation to low protein intake, and (2) the obligatory nitrogen losses.

The human body can conform to low nutrient intakes without adverse consequences within certain limits [48]. As already stated, body proteins undergo an extensive turnover (continuous breakdown and synthesis); amino acids derived from protein breakdown are recycled for new synthesis or inexorably oxidized or seized (Figure 2). In the case of reduced protein (or AA) supply, a rapid and more efficient re-utilization of endogenous AAs occur with a reduced rate of EAA oxidization. After some time, a reduction in protein synthesis and breakdown occurs. With these mechanisms, the body adapts, until certain limits, to a reduced AA intake, by reducing nitrogen losses. After the so-called “obligatory nitrogen losses”, despite continuing a protein-free regimen, it is not possible to further limit nitrogen losses [49,50,51,52]. The obligatory nitrogen losses are measurable in individuals receiving a protein-free diet after a brief period of adaptation (usually 4–5 days). These losses were estimated, as shown in Table 3 [53]:

The total obligatory nitrogen losses are ~56 mg/Kg BW/day, which, converted with factor 6.25, represent a protein loss of ~0.35 g/Kg BW/day. These appear to be quite uniform in healthy adults, even in different countries where daily protein intake may widely vary [43]. The regression of the nitrogen balance on the protein intake in healthy people estimates the minimum protein requirement. Usually, nitrogen balance is measured after a short period of adaptation to specific protein intake. The lowest protein intake corresponding to a nitrogen equilibrium represents the minimum protein requirement. Several studies measuring the nitrogen balance at different levels of protein intake in healthy subjects, fixed the median of the minimum protein requirement at ~0.66 g/Kg BW/day, with considerable inter-individual variations. The estimate for the 97.5th percentile (taking into account 2 SD on the median) provides a daily dietary protein allowance of ~0.83 g/Kg BW/day by taking into account the inter-individual variability, therefore representing a safety amount for almost all of the adult population [43]. The daily protein requirement can vary in different physiological and pathological conditions. In the first case, the European Food Safety Authority [54] suggests a daily dietary allowance of 0.83 g/Kg BW/day for adults, while for infants, children, and adolescents, a value between 0.83 and 1.31 g/Kg BW/day depending on age. For pregnant women, an additional intake of 1, 9, and 28 g, respectively, for the first, second, and third trimester of pregnancy is necessary, whereas for breastfeeding women an additional intake of 19 g per day in the first six months of breastfeeding and 13 g in the following period. It is also recommended a surplus of protein in the diet for individuals who regularly exercise. For example, the recommended daily intake for endurance training is 1.2 to 1.4 g/Kg BW/day, while for strength training it is 1.6 to 1.8 g/Kg BW/day.

## 6. Amino Acid Requirement in Parenteral Nutrition

The step from protein requirement assessment to AA supply in PN must take into account the formation of the peptide bond, a dehydration reaction. During protein synthesis, the bond of each two AAs (i.e., the creation of peptide bond) releases a water molecule; thus, 100 g of AA provides a protein substrate of ~83 g [55]. For this reason, a protein requirement of 0.83 g/Kg BW/day must correspond to an AA supply of ~1 g/Kg BW/day. For the same reason, 100 g of AA, once transformed into proteins, do not provide 400 Kcal and 16 g of nitrogen, but 340 Kcal and 13.3 g of nitrogen [55].

Protein synthesis and breakdown require energy and are therefore sensitive to a reduction or increase in energy intake. Indeed, there is a strong relationship between protein requirement and energy intake [56,57]. As a consequence, energy intake (mostly provided by carbohydrates and lipids) influences the needs of AAs and vice versa. The amount of energy supplied with carbohydrates and/or lipids affects the nitrogen balance, the use of AAs (for protein synthesis or energy production), and the urinary excretion of nitrogen [58]. Thus, when calculating the AA requirement, it is necessary to consider the body’s energy balance. Adequate energy supply would allow the use of adequate AA supply for exclusive protein synthesis use, reducing urinary nitrogen excretion, and increasing the nitrogen balance. Instead, an inadequate energy supply will force the body to use part of AAs to produce energy with a consequent increase in urinary nitrogen excretion and reduction of nitrogen balance. An adequate energy supply, associated with an AA excess, would meet the body protein synthesis requirements. The over-plus of AA will be oxidized in the meantime, with the production of energy excess, increase in urinary nitrogen excretion, and nitrogen balance.

In summary, an inverse correlation exists between AA requirements and energy intake: for higher AA amounts, less energy is required, and vice-versa. The idea of the non-protein calorie/nitrogen ratio (NPC/N) relies upon the rule that energy supply is needed to prevent oxidation of amino acids [58]. NPC/N ranges from 125 to 225 Kcal/g N for non-stressed PN patients [59]. According to the American Society for Parenteral and Enteral Nutrition guidelines, the best NPC/N ratio is about 70:1 to 100:1 for critically ill patients [60], reduced to 30:1 to 50:1 for obese, critically ill patients [28]. As the standard PN mixtures have a high NPC/N, additional protein supplements may be required to provide an adequate amount of AAs.

The AA requirement for PN patients depends on nutritional status (mild, moderate, or severe malnutrition), metabolism (normal, increased or elevated), and underlying clinical condition. This requirement can be estimated by studying nitrogen balance, measuring urinary urea excretion. As already mentioned, nitrogen losses occur through urinary excretion, feces, and a miscellany of other secretions. In clinical practice, the last two losses being difficult to measure, a constant factor has been assigned. Urine nitrogen losses are due to the excretion of urea (which represents about 80%), ammonia, uric acid, creatinine, nitrates, amino acids, and other nitrogen compounds. Since urinary urea is routinely measured, some formulas using only urea dosage have been developed to estimate total nitrogen losses. The best-known formula used to estimate the total nitrogen loss is [61,62,63]

Total Nitrogen Loss = g Urinary Urea Nitrogen (24 h excretion) + 4(1)

The constant factor accounts both for non-urea urinary nitrogen losses (e.g., ammonia, uric acid, creatinine; about 2 g of nitrogen) and nitrogen losses through feces and other pathways (e.g., sweat, sebum, peeling of the skin; about the other 2 g of nitrogen) [64]. Considering that 1 g of urea contains 0.46 g of N, then the formula can be written as

Total Nitrogen Loss = g Urinary Urea (24 h excretion) × 0.46 + 4(2)

The severity of the disease could alter total nitrogen losses; in these cases, the assumption of a 4-g non-urea nitrogen component to urine and insensible losses may not be reliable. As a consequence, this formula may not be suitable for multiple clinical conditions such as severely burned patients, those with significant gastrointestinal losses, and critically ill patients. Thus, more specific formulas may be used [65]. 

To reach a positive nitrogen balance, and therefore an anabolic state, it is necessary to provide a dose of AAs higher than nitrogen losses. However, it is recommended not to exceed the suggested limits by taking into account either the underlying disease and the capacity of utilization and metabolization of the supplied AA. For example, in adult patients without organ failure, the suggested limit of ~2.4 g AA/Kg BW/day corresponds to the maximum infusion rate for AA solution of ~0.1 g AA/Kg BW/h (obtained by dividing the maximum daily dose by 24 h). Consequently, the maximum infusion rate of a prescribed daily dose of AA can be calculated with

BW (Kg) × 2.4 (g AA/Kg BW/day)/24 (h) = BW (Kg) × prescribed daily dose (g AA/Kg BW/day)/time of infusion (h)(3)

Time of infusion (h) = 10 × prescribed daily dose(4)

For instance, for a prescribed dose of 1 g AA/Kg BW/day, the infusion time will be equal to 10 h. In the case of an all-in-one mixture, the estimate of the maximum infusion rate must consider the presence of all the other macronutrients and the total volume of the mixture.

## 7. Amino Acid Requirement for Adult Patients without Organ Failure

AA and energy requirements vary according to the metabolic state (assessed by estimating nitrogen losses and resting metabolic rate) and the PN goal (muscle mass loss prevention or restoration). Normal or moderate nitrogen losses indicate a normal or slightly altered catabolic state. In both cases, the suggested AA requirement is 0.8 to 1 g AA/Kg BW/day with a daily energy supply ranging between 20 and 25 Kcal/Kg BW, based on the physical activity [1,66]. In patients with increased nitrogen loss, the suggested requirement is 1.2 to 1.5 g AA/Kg BW/day [67]. In those with elevated losses, it can be up to 2.5 g AA/Kg BW/day, representing the maximum daily limit [67]. In critically ill patients, the daily AA requirement is always high due to protein loss through fistulas, ulcers, drug treatments, increased catabolism (trauma or sepsis). In these cases, 2 to 2.5 g AA/Kg BW/day can improve the ICU patient’s outcome [67]. However, the full PN has to be gradually reached between the first 3 to 7 days of ICU admission [67]. The addition of glutamine to PN mixtures is suggested for critically ill patients with trauma (0.2–0.3 g glutamine/kg BW/day) or burns >20% body surface area (0.3–0.5 g glutamine/kg BW/day) [63]. On the other hand, in unstable ICU patients, parenteral glutamine is not indicated, particularly with hepatic or renal failure [67].

Energy and AA requirements in ICU patients are extremely variable during the different phases of hospitalization. The energy expenditure (EE) should be measured by indirect calorimetry due to the inaccuracy of the predictive EE equations. In any case, in the early period of acute illness, energy supplementation starts with 70% of the total EE requirement, to increase up to 80% to 100% of measured EE after three days [67].

## 8. Adult Patients with Cancer

Cancer patients face a high risk of malnutrition due to the underlying clinical condition, radio/chemo-treatment, and inadequate nutritional intake. The consequence is a significant loss of body weight and muscle mass, with worsening of performance status, quality of life, and outcome. For this reason, in these patients, the nutritional status has to be accurately screened, monitored, and eventually treated, starting from diagnosis and all through the clinical follow-up. PN in cancer patients is based primarily on the inaccessibility of the GI tract, treatment effect on nutrient intakes, and patient compliance with oral or enteral nutrition. The most recent guidelines suggest an AA requirement of 1.2 to 1.5 g of AA/Kg BW/day with an energy intake of 25 to 30 Kcal/Kg BW/ day [68]. The use of standard formulas to estimate EE often leads to inaccurate results due to the specific metabolic alterations for the different types of cancer; also in this case, it would be more appropriate to use indirect calorimetry for EE assessment. The addition of anti-catabolic and anti-inflammatory nutrients to PN mixtures such as a higher content of EAA and leucine, as well as omega-3 fatty acids, seems promising. However, further studies are required to confirm real improvements in clinical outcomes.

## 9. Liver Failure

In acute liver failure, the most important consequences are the reduced production of hepatic glucose and a substantial increase in protein catabolism, with an over-production of amino acids and ammonium. In particular, the plasma AA levels are increased, and their pattern is altered, showing a relative decrease in BCAAs and an increase in aromatic AAs, methionine, and tryptophan. In patients with hyperacute liver failure, severe hepatic encephalopathy, and elevated ammonium levels, the supply of AAs can be delayed for 24 to 48 hours until the reduction of ammonium levels. As already reported [27,28], standard AA solutions can be infused in hepatic encephalopathy up to grade II. Liver-adapted AA solutions may be considered for the grade III and IV of encephalopathy in the patients refractory to conventional management. There is no reliable information on the nutritional needs of patients with acute liver failure; thus, individualized nutrition should be preferred, using the same evaluation methods used for critically ill patients. Suggested ranges of needs for compensated liver patients are 1.2 to 1.5 g AA/Kg BW/day for proteins and 30 to 35 Kcal/Kg BW /day for energy [69].

## 10. Chronic Intestinal Failure

The nutrient requirement in adult patients with intestinal insufficiency, and particularly patients with short bowel syndrome (SBS) and ileostomy/jejunostomy, must be individually assessed and adapted to the global clinical conditions, nutritional status, organ functions, input/output balance, amongst others. Most SBS patients need a customized macro- and micronutrient supply; for this reason, they must be followed up exclusively at third level artificial nutrition centers endowed with a hospital pharmacy to compound personalized parenteral nutrition mixtures. The AA requirement of stable SBS patients is often met by an AA supply of 0.8 to 1.4 g/kg BW/day, with a balanced energy intake to allow the best use of nitrogen. The evaluation of energy needs can be carried out with the methods used for the general population [70].

Patients with inflammatory bowel disease (IBD) on the active phase need an AA supply between 1.2 to 1.5 g AA/Kg BW/day. During the disease remission, AA and energy supply equal that recommended for the general population [71].

## 11. Renal Failure

Substitutive treatment in patients with acute or chronic renal failure causes a loss of a certain dose of AAs, promotes protein catabolism, and reduces protein synthesis speed [29]. Consequently, a supply of 1.2 to 1.5 g (in hemodialysis), 1.2 to 1.4 g (in peritoneal dialysis), or 1.5 to 2.5 g AA/Kg BW/day (in continuous renal replacement therapy) could be appropriate [66].

In patients with moderate chronic renal failure in conservative therapy, an AA intake up to 0.8 to 1.2 g/Kg BW/day until a glomerular filtration rate of 30 mL/min is suggested. In the case of lower filtrate, AA intake must be reduced to ~0.6 g AA/Kg BW/day [29], and the patient should also be nutritionally monitored. For patients with acute and chronic renal failure, energy intake is around 25 to 30 Kcal /Kg BW/day [66].

## 12. Pediatric Patients

The protein (or AA) requirements in infants and children are higher than in adulthood and change according to age ranges: preterm born (age <37 weeks), full-term born (37–41 weeks), infants and children between one month and three years, children aged 3-12 years, adolescents (12 < age < 18 years). The aim of PN in preterm born is to achieve a regular postnatal growth rate. In detail, on the first postnatal day, the AA supply should be at least 1.5 g/Kg BW/day [31], with an energy intake of 45–55 Kcal/Kg BW/day [72]. From the second day of life on, the AA supply should be 2.5 to 3.5 g/Kg BW/day [31] with an energy intake of 90 to 120 Kcal/Kg BW/day [72]. In full-term infants, an AA provision should be 1.5 to 3.0 g/Kg BW/day [31] with an energy supply of 75 to 85 Kcal/Kg BW/day [72]. In infants and children from 1 month to 3 years, the AA and energy requirements are 1.0 to 2.5 g/Kg BW/day and 65 to 85 Kcal/Kg BW/day, respectively [31,72]. In children aged 3–12 years, an AA intake of 1.0 to 2.0 g/Kg BW/day [31] can be considered, with an energy intake of 55 to 75 Kcal/Kg BW/day [72]. For adolescents, an AA intake of 1.0 to 2.0 g/Kg BW/day [31] with an energy intake of 30–55 Kcal /Kg BW/day is suggested [72].

## 13. Conclusions

AA supply has a pivotal role in the maintenance or recovery of muscle mass in malnourished PN patients. The daily requirement of AA depends on nutritional status, age, weight, sex, energy balance, and underlying disease. Despite a wide variety of commercially available all-in-one PN mixtures, often, patient nutritional needs are not fully met, both for the AA/energy ratio and also for other nutrients and electrolytes. When available, personalized PN mixtures meet the specific nutritional needs of single patients. However, because most stock AA solutions have an unsatisfactory amount of EAAs, even in personalized mixtures, the AA profile could be far from the ideal. An AA solution containing a more substantial amount of EAAs, including cysteine and tyrosine, could represent an improvement over the existing products. Concerning the nutritional requirements, the scientific guidelines represent the basis for establishing energy and AA requirements; however, clinical nutrition specialists should refine AA and energy supplies according to individual needs.

## Figures and Tables

**Table 1:** EAA percentage of total AAs in several commercially available IV standard amino acid solutions.

	Current Commercially Available IV Standard Amino Acid Solutions						
	Aminoven 10%	Sintamin 10%	Isopuramin 10%	Parentamin 10%	Amixal 10%	Freamine 10%	Travasol 10%
**EAA percentage of total AAs**	44.4	52.4	74.9	58.4	41.2	50.8	49.3

AA: amino acid; EAA: essential amino acid; IV: intravenous.

**Table 2:** EAA percentage of total AAs in several IV complete, all-in-one parenteral mixtures that are commercially available in Italy.

	Current Commercially Available IV Complete All-in-one Parenteral Mixtures				
	Periven Kabiven	Krinuven Smofkabiven Aminomix	Nutriplus Nutrispecial Nutriperi Basalflex Periflex Plusflex Specialflex	Clinimix Oliclinomel	Olimel
**EAA percentage of total AAs**	51.2	43.3	48.6	45.7	51.1

AA: amino acid; EAA: essential amino acid; IV: intravenous.

**Table 3:** Nitrogen losses by type of excretion and per kg of body weight in healthy adult patients.

	Nitrogen Losses	% of nitrogen Losses
**Urine**	~ 36 mg/Kg BW/day	65%
**Feces**	~ 12 mg/Kg BW/day	21%
Other pathways *	~ 8 mg/Kg BW/day	14%

* Sweat, sebum, desquamation of the skin, nails, hair, saliva.
